# Feasibility of Early Surgical Treatment for Adolescent Patients with Prolactinoma: A Case Report and Literature Review

**DOI:** 10.3390/medicina60081345

**Published:** 2024-08-19

**Authors:** Yu-Hung Tsai, Chi-Ruei Li, Yu-Ting Wang, Se-Yi Chen

**Affiliations:** 1 Department of Neurosurgery, Chung Shan Medical University Hospital, Taichung 40201, Taiwan; ddyuhong@gmail.com; 2 Department of Neurosurgery, Neurological Institute, Taichung Veterans General Hospital, Taichung 40705, Taiwan; 3 Department of Anatomical Pathology, Chung Shan Medical University Hospital, Taichung 40201, Taiwan; cshy1399@csh.org.tw; 4 School of Medicine, College of Medicine, Chung Shan Medical University, Taichung 40201, Taiwan

**Keywords:** prolactinoma, pituitary adenoma, pituitary apoplexy, menarche, fertility

## Abstract

*Background and Objectives*: Prolactinomas are the most common pituitary adenomas, comprising 30–50% of such tumors. These adenomas cause hyperprolactinemia, leading to decreased fertility, reduced energy and libido, and galactorrhea. Diagnosing and treating prolactinomas in adolescents present unique challenges, as symptoms may be confused with age-related developmental variations. This case report explores the outcomes of early surgical intervention in an adolescent with a prolactinoma. *Materials and Methods*: A 14-year-old female presented delayed menarche and absent pubertal development. Initial evaluation revealed hyperprolactinemia (228.37 ng/mL) with normal estradiol levels. Initial management through observation was adopted, but persistent amenorrhea and severe headaches prompted further investigation. Magnetic resonance imaging revealed a cystic pituitary mass with apoplexy. Due to concerns regarding delayed puberty and the need for rapid normalization of prolactin levels, the patient underwent transsphenoidal surgery. *Results:* After operation, prolactin levels normalized, menarche occurred within three months, and secondary sexual characteristics developed within eight months. Pathology confirmed a pituitary adenoma with a high Ki-67 index (15%). *Conclusions*: Early surgical intervention for prolactinomas in adolescents can achieve successful biochemical remission and resolution of endocrine symptoms. Adolescents, particularly those with a high Ki-67 index and potential resistance to dopamine agonists, may benefit from prompt surgical management, resulting in improved clinical outcomes and complete tumor resection.

## 1. Introduction

Prolactinomas are the most common pituitary adenomas, accounting for approximately 30% to 50% of all pituitary adenomas [1,2]. The clinical presentation associated with excessive prolactin secretion (typically exceeding 200 ng/mL) includes decreased fertility, reduced energy and libido, and galactorrhea in men and premenopausal women. Additionally, these tumors can compress the optic nerves and optic chiasm, leading to visual field and visual acuity deficits.

Adolescents with prolactinomas are a relatively rare group [3,4], and their endocrinological symptoms may often be missed or underestimated due to age-related factors. Consequently, the suitability of applying adult treatment strategies for adolescent patients remains uncertain [5,6,7,8]. There is also significant concern regarding hormonal recovery and future fertility in adolescents. The risk of pan-hypopituitarism following surgery complicates treatment decisions, making it crucial to consider the unique needs and potential risks in this younger population.

Medical treatment with dopamine agonists (DAs) is the first-line therapy for prolactinomas, aiming to reduce tumor size and restore gonadal function and fertility. Surgical tumor resection is typically reserved for emergencies, such as pituitary apoplexy with acute visual loss. Surgical management may also be preferred for patients desiring future fertility or those who cannot tolerate the side effects of DAs, which include nausea, headache, hypotension, and psychotic symptoms [9,10]. Despite the potential benefits, there are few published cases of surgical treatment in adolescent patients with prolactinomas [11,12].

In this case report, we present an adolescent female patient with hyperprolactinemia and absent menarche, who also experienced severe headaches due to tumor-related apoplexy. Following immediate transsphenoidal surgery, the prolactin levels of the patient rapidly normalized, her first menarche occurred within three months, and secondary sexual characteristics, including breast development, were observed after eight months. This case report aims to explore strategies for managing adolescent patients with prolactinomas, particularly whether early surgical intervention leads to better clinical outcomes.

## 2. Case Description

A 14-year-old female patient, with no history of systemic or congenital diseases and not on any long-term medication, presented with delayed menarche and absent pubertal development. The patient reported no significant weight or height gain over the past six months. Three months earlier, she had sought help at a gynecology clinic. The initial sonographic evaluation revealed no uterine abnormalities, and blood tests showed hyperprolactinemia (228.37 ng/mL) with normal estradiol levels (39.9 pg/mL). Observation was initially adopted; however, over the following three months, the patient remained amenorrheic and began experiencing intermittent severe headaches over the four weeks preceding admission. Additional hormone evaluations revealed normal levels of growth hormone (0.106 ng/mL), thyroid stimulating hormone (2.242 µIU/mL), and adrenocorticotropic hormone (41.4 pg/mL). Consequently, she was referred to our neurosurgery department for further evaluation and management.

**Figure 1:**
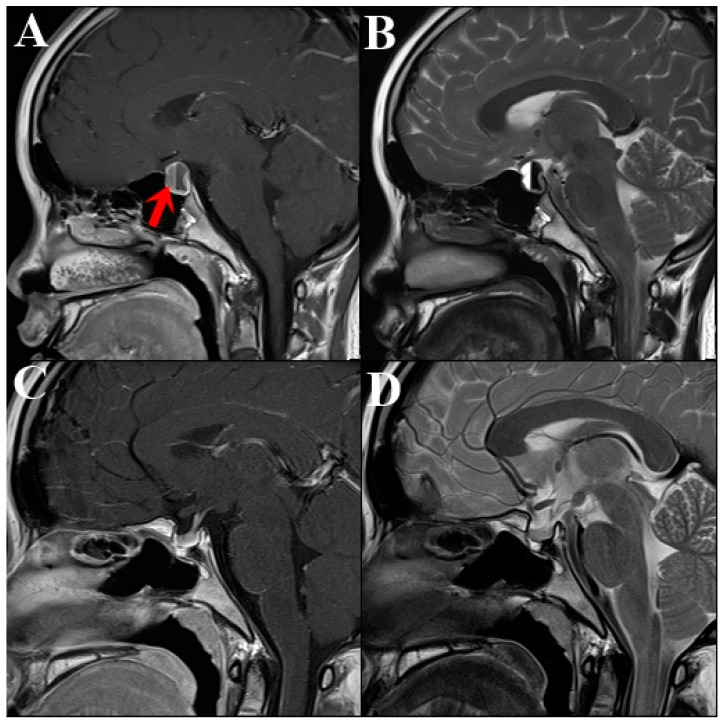
The pre-operative brain MRI. (**A**) T1-weighted image with contrast demonstrating a well-defined cystic mass (1.3 × 1.0 × 1.0 cm) arising from the pituitary fossa (red arrow), with suprasellar extension causing mild mass effect and slight indentation of the optic chiasm. (**B**) T2-weighted image revealing fluid accumulation within the mass, suggestive of hematoma. These findings are consistent with pituitary adenoma-induced pituitary apoplexy with hemorrhage. (**C**,**D**) Eight months post-operative brain MRI. Both T1-weighted with contrast and T2-weighted images showing no abnormal finding in the residual pituitary gland, pituitary stalk, and optic chiasm. MRI, magnetic resonance imaging.

The prolactin levels of the patient remained abnormally high (290.69 ng/mL), with extremely low estradiol levels (<10 pg/mL). Brain magnetic resonance imaging (MRI) identified a well-defined cystic mass measuring 1.3 × 1.0 × 1.0 cm in the pituitary fossa, with a fluid–fluid level and suprasellar extension, suggesting a pituitary adenoma with apoplexy (Figure 1). Automated perimetry was conducted before surgery, revealing no significant visual defects in either eye. After thoroughly discussing the advantages and disadvantages of treatment options, including DAs and surgery, the patient and her family opted for surgery due to concerns about delayed puberty and the necessity of a quicker normalization of prolactin levels.

**Figure 2:**
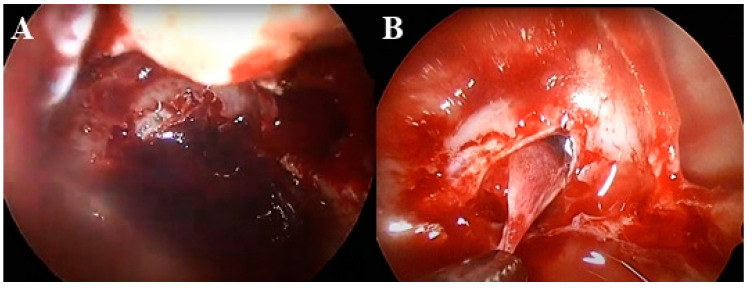
Intraoperative image under the endoscope. (**A**) Removal of dark-red hematoma following dura incision. (**B**) Visualization of a soft, gummy tumor after removal of the hematoma.

**Figure 3:**
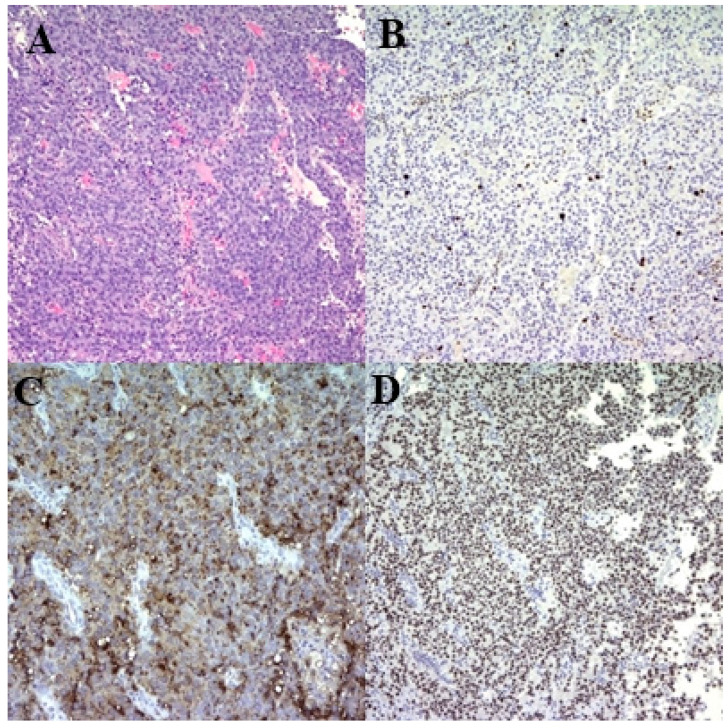
Histopathological characteristics of the lactotroph pituitary neuroendocrine tumor (PitNET). (**A**) H&E stain showing diffuse, mixed solid and trabecular patterns of uniform cells with chromophobic cytoplasm. (under H&E stain). (**B**) Immunohistochemical stain for Ki-67 revealing a proliferation index of 15%, indicating potential aggressiveness of the lactotroph tumor. (**C**) Immunohistochemical stain for prolactin demonstrating cells with a paranuclear globular Golgi-like pattern. (**D**) Immunohistochemical stain for Pit-1 confirming positivity in tumor cells. PitNET, pituitary neuroendocrine tumor; H&E, hematoxylin and eosin.

We conducted an endonasal transsphenoidal adenomectomy. Intraoperatively, a dark-red hematoma was removed following dura incision. A soft, gummy tumor was identified beneath the hematoma and resected using a Hardy curette (Figure 2). The pituitary gland and stalk were well preserved during the procedure. Postoperatively, prolactin levels rapidly normalized (5.2 ng/mL). After surgery, the prolactin level rapidly returned to the normal range (5.2 ng/mL), with no significant changes in other pituitary hormones: growth hormone (0.125 ng/mL), thyroid stimulating hormone (1.338 µIU/mL), and adrenocorticotropic hormone (39.5 pg/mL). Pathological examination confirmed a pituitary adenoma with immunohistochemical staining revealing a Ki-67 index of 15%, indicating a potentially aggressive lactotroph tumor (Figure 3).

During the postoperative period, increased urine output of up to 4500 c.c./day was noted on day 2, initially suggesting diabetes insipidus. However, the patient maintained normal serum sodium levels (136 mmol/L). On day 5, she developed progressing nausea, vomiting, and headaches, and she experienced a self-limited seizure. Subsequently, her serum sodium levels dropped to 113 mmol/L. Intensive monitoring of sodium levels, urine output, and blood osmolality every four hours revealed that the patient alternated between syndrome of inappropriate antidiuretic hormone secretion (SIADH) and cerebral salt wasting. Electrolyte and fluid imbalances were managed with either 3% sodium chloride infusion, fluid supplementation, or fluid restriction based on laboratory data and fluid status. The patient’s sodium levels and clinical symptoms gradually stabilized and completely normalized by day 7. She was discharged in stable condition without the need for cortisol supplementation, and there were no further episodes of hyponatremia after discharge.

**Figure 4:**
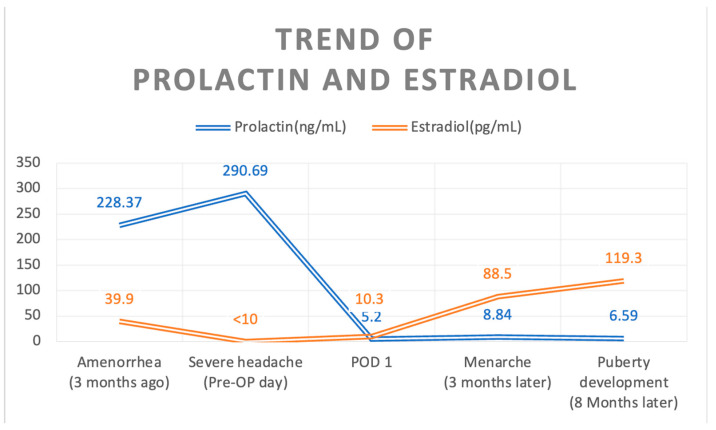
Trend of prolactin and estradiol levels over time. The figure illustrates hyperprolactinemia at 228.37 ng/mL, which peaked at 290.69 ng/mL, and normal estradiol levels at 39.9 pg/mL, which decreased to undetectable levels in the three months preceding surgery. Post-surgery, prolactin levels quickly normalized and remained stable within the normal range over the subsequent eight months. Estradiol levels gradually increased following surgery.

Three months later, prolactin and estradiol levels remained within the normal range, and the patient experienced her first menarche. Eight months after the surgery, she exhibited secondary sexual characteristics, including breast development and a regular menstrual cycle. A follow-up brain MRI eight months after surgery revealed no abnormalities in the residual pituitary gland, pituitary stalk, or optic chiasm (Figure 1C,D). Consistent prolactin and estradiol levels were maintained during outpatient follow-up visits (Figure 4). 

## 3. Discussion

Prolactinoma in pediatric or adolescent patients is relatively rare. Unlike in adults, endocrinological symptoms in younger patients can often be mistaken for age-related developmental variations. For instance, delayed menarche in an adolescent girl might initially be attributed to normal developmental variation rather than a prolactinoma, potentially delaying diagnosis and treatment. The appropriateness of applying adult management guidelines to adolescent patients remains controversial due to these unique characteristics. Common hormone deficiencies associated with pituitary apoplexy include ACTH, TSH, gonadotropin (LH and FSH), GH, prolactin, and ADH deficiency, which may lead to fatigue, weakness, weight gain or loss, irregular or absent menstrual periods, infertility, growth retardation, diabetes insipidus, and secondary life-threatening situations. Therefore, early surgical intervention is important in patients with pituitary apoplexy.

In this case report, we present an adolescent patient with a prolactinoma whose primary symptom was delayed puberty, complicated by pituitary apoplexy. Following immediate surgical treatment, the prolactin levels of the patient rapidly normalized, and the tumor-related delayed puberty resolved without any adverse events.

Pharmacological management with dopamine agonists (DAs) is generally the first-line treatment for prolactinomas [13]. Surgical resection is typically reserved for patients with neurological symptoms, such as visual disturbances, or those intolerant to the side effects of DAs. Drug discontinuation due to side effects, such as nausea, vomiting, mild orthostatic hypotension, and headaches, occurs in 3% to 12% of cases [14,15]. Although psychotic side effects are rare, physicians must monitor for impulse control disorders, depression, mania, and other types of psychosis [16,17,18].

Miermeister et al. [19] proposed a new cut-off value for the Ki-67 index (>4%) as the best marker for diagnosing atypical pituitary adenomas. Thapar et al. [20] demonstrated that a high Ki-67 labeling index is typically found in prolactinomas, supporting the notion that a high Ki-67 index is indicative of a more proliferative and potentially more aggressive tumor. Our patient showed a Ki-67 index of 15%, which is significantly higher than the average range reported in the previous literature (0.5–2.64%) [21,22,23], suggesting a highly aggressive tumor. This Ki-67 index far exceeding the cut-off value falls into a category of atypical adenomas, which are known for their aggressive nature and poorer prognosis. The literature also suggests that younger patients tend to exhibit higher Ki-67 indices compared to older patients, indicating a greater proliferative potential and higher risk of aggressive tumor behavior [23,24].

Given the well-established correlation between dopamine agonist (DA) resistance and tumor invasiveness [23,25,26,27,28], this is especially pertinent for adolescent patients with prolactinomas. In this demographic, a high Ki-67 index not only indicates potential malignancy but also indicates a likelihood of resistance to traditional medical treatments like DAs. This underscores the potential advantages of prioritizing surgical intervention as the initial approach.

First-line surgical intervention might be more effective for younger patients with prolactinomas due to their higher Ki-67 indices and associated challenges in managing more aggressive and DA-resistant tumors. Early surgery can potentially mitigate the risks associated with delayed or inadequate response to medical therapy, thereby improving the overall prognosis. Additionally, surgical treatment often results in a more rapid normalization of hyperprolactinemia compared to DA therapy alone, providing faster symptomatic relief and reducing the risk of long-term complications associated with elevated prolactin levels [29].

Rescue surgical resection of DA-resistant prolactinomas is frequently incomplete due to fibrosis induced by prior DA therapy [30,31]. The presence of fibrosis during surgery is recognized as a negative predictor for achieving complete biochemical remission [31,32,33]. Therefore, early surgery in these cases may enhance remission rates and the likelihood of successful complete tumor resection [34].

However, surgical intervention carries common risks, such as cerebrospinal fluid (CSF) rhinorrhea, meningitis, electrolyte and fluid imbalance, and damage to the pituitary gland, cerebral vessels, and the visual nerve tracts during pituitary surgery. Some patients hesitate to undergo pharmacological treatment due to the need for long-term treatment, a rebound effect after drug discontinuation, side effects, and potential permanent neurological deficits caused by delayed surgery. Given the well-established correlation between DA resistance and tumor invasiveness, and the higher trend of the Ki-67 index in younger patients indicating potential DA resistance, we propose that surgical intervention should be preferred over pharmacological intervention as the first-line treatment in patients with progressive neurological symptoms, severe pharmacological side effects, and young age. However, more cases involving younger patients are needed to establish better subgroup recommendations for choosing surgery as the first-line treatment.

A limitation of our case report is the scarcity of similar reports or case series involving adolescent patients with prolactinomas, which restricts the generalizability of our findings. Furthermore, caution is warranted against overinterpreting conclusions drawn from a single case. Therefore, additional studies encompassing larger cohorts and comparative research are essential to corroborate the feasibility and benefits of early surgical intervention.

This report describes the case of an adolescent with a prolactinoma who underwent prompt surgical resection, resulting in the normalization of prolactin levels and the resolution of tumor-related delayed puberty. Drawing from a literature review, we specifically highlight the potential benefits of early surgical management for adolescent patients, particularly those at higher risk of DA resistance, aiming to optimize clinical outcomes and achieve complete tumor resection.

## 4. Learning Points

- Adolescents with prolactinomas may present with atypical symptoms that can mimic normal developmental variations, necessitating a high index of suspicion for accurate diagnosis.
- Prompt surgical intervention in adolescents with prolactinomas can lead to rapid normalization of prolactin levels and resolution of associated endocrine dysfunction, such as delayed puberty.
- High Ki-67 indices in prolactinomas, indicating increased proliferative activity, may predict resistance to dopamine agonist therapy and highlight the potential role of early surgical management.
- Postoperative outcomes in adolescent patients, including rapid onset of menarche and development of secondary sexual characteristics, underscore the effectiveness of surgical intervention in restoring normal hormonal function.

## 5. New Findings

- This case report emphasizes the effectiveness of early surgical intervention in achieving biochemical remission and resolving endocrine symptoms in adolescent patients with prolactinomas, particularly those with high Ki-67 indices.
- The study highlights the unique challenges and successful outcomes associated with early surgical management in adolescents, contributing valuable insights into treatment strategies for this age group.

## Data Availability

Restrictions apply regarding the availability of these data, as they are not publicly available. However, the data are available from the corresponding author upon reasonable request.

## List of Abbreviations

DA	Dopamine agonists
MRI	Magnetic resonance imaging
